# (Acetato-κ*O*)bis­(1,10-phenanthroline-κ^2^ *N*,*N*′)copper(II) acetate hepta­hydrate

**DOI:** 10.1107/S1600536811009676

**Published:** 2011-03-19

**Authors:** Buqin Jing, Lianzhi Li, Jianfang Dong, Tao Xu

**Affiliations:** a College of Chemistry and Chemical Engineering, Shanxi Datong University, Datong, Shanxi 037009, People’s Republic of China; b School of Chemistry and Chemical Engineering, Liaocheng University, Shandong 252059, People’s Republic of China; c Department of Material Science, Shandong Polytechnic Technician College, Shandong 252027, People’s Republic of China

## Abstract

In the title complex, [Cu(CH_3_CO_2_)(C_12_H_8_N_2_)_2_](CH_3_CO_2_)·7H_2_O, the central Cu^II^ ion is five coordinate, being bound to four N atoms from two 1,10-phenanthroline ligands and one O atom from an acetate anion in a strongly distorted square-pyramidal configuration. Hydrogen-bonded water mol­ecules and an uncoordinated acetate anion form a two-dimensional polymeric structure parallel to (010). The cations are linked to this layer *via* O—H⋯O hydrogen bonds between one of the water mol­ecules and the coordinated acetate anion.

## Related literature

For the structures of similar five-coordinate copper(II) complexes with 1,10-phenanthroline and carboxyl­ate anions, see: Tu *et al.* (2008 ▶); Xu *et al.* (2008 ▶).
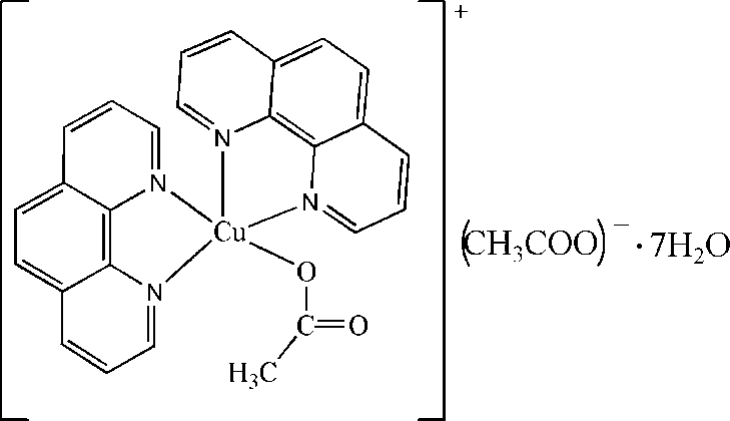


## Experimental

### 

#### Crystal data

[Cu(C_2_H_3_O_2_)(C_12_H_8_N_2_)_2_](C_2_H_3_O_2_)·7H_2_O
*M* *_r_* = 668.15
Triclinic, 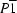

*a* = 8.764 (4) Å
*b* = 12.307 (5) Å
*c* = 15.739 (7) Å
α = 103.257 (7)°
β = 102.243 (7)°
γ = 97.606 (7)°
*V* = 1585.2 (12) Å^3^
*Z* = 2
Mo *K*α radiation
μ = 0.75 mm^−1^
*T* = 298 K
0.42 × 0.38 × 0.32 mm

#### Data collection

Bruker SMART 1000 CCD area-detector diffractometer
Absorption correction: multi-scan (*SADABS*; Sheldrick, 1996 ▶) *T*_min_ = 0.728, *T*_max_ = 0.795
8364 measured reflections
5570 independent reflections
3220 reflections with *I* > 2σ(*I*)
*R*_int_ = 0.040

#### Refinement

*R*[*F* ^2^ > 2σ(*F* ^2^)] = 0.057
*wR*(*F* ^2^) = 0.130
*S* = 0.95
5570 reflections
399 parameters
52 restraints
H-atom parameters constrained
Δρ_max_ = 0.42 e Å^−3^
Δρ_min_ = −0.52 e Å^−3^

### 

Data collection: *SMART* (Bruker, 2007 ▶); cell refinement: *SAINT* (Bruker, 2007 ▶); data reduction: *SAINT*; program(s) used to solve structure: *SHELXS97* (Sheldrick, 2008 ▶); program(s) used to refine structure: *SHELXL97* (Sheldrick, 2008 ▶); molecular graphics: *SHELXTL* (Sheldrick, 2008 ▶) and *DIAMOND* (Brandenburg, 1999 ▶); software used to prepare material for publication: *SHELXTL*.

## Supplementary Material

Crystal structure: contains datablocks global, I. DOI: 10.1107/S1600536811009676/gk2351sup1.cif

Structure factors: contains datablocks I. DOI: 10.1107/S1600536811009676/gk2351Isup2.hkl

Additional supplementary materials:  crystallographic information; 3D view; checkCIF report

## Figures and Tables

**Table 1:** Selected bond lengths (Å)

Cu1—N3	1.988 (3)
Cu1—N1	1.989 (3)
Cu1—O1	2.001 (3)
Cu1—N4	2.051 (3)
Cu1—N2	2.191 (4)

**Table 2:** Hydrogen-bond geometry (Å, °)

*D*—H⋯*A*	*D*—H	H⋯*A*	*D*⋯*A*	*D*—H⋯*A*
O5—H29⋯O3	0.85	1.90	2.738 (5)	170
O6—H31⋯O5	0.85	1.92	2.768 (5)	176
O7—H33⋯O4	0.85	1.96	2.797 (5)	167
O7—H34⋯O8	0.85	1.91	2.760 (5)	173
O8—H36⋯O2	0.85	1.86	2.710 (4)	173
O9—H37⋯O8	0.85	1.98	2.826 (5)	174
O10—H40⋯O11	0.85	2.23	3.070 (5)	168
O11—H42⋯O9	0.85	1.89	2.714 (5)	164
O5—H30⋯O10^i^	0.85	2.03	2.819 (5)	155
O6—H32⋯O4^i^	0.85	1.94	2.778 (5)	169
O9—H38⋯O6^i^	0.85	1.94	2.737 (6)	156
O10—H39⋯O4^ii^	0.85	1.87	2.702 (5)	164
O11—H41⋯O7^ii^	0.85	1.88	2.724 (5)	170
O8—H35⋯O11^iii^	0.85	1.97	2.796 (5)	165

Symmetry codes: (i) 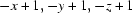; (ii) 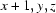; (iii) 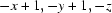.

**Fig. 1.**
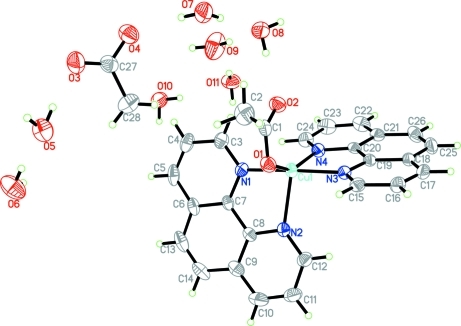
The asymmetric unit of the title compound drawn with 30% probability displacement ellipsoids.

**Fig. 2.**
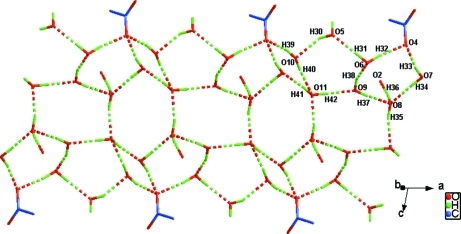
Fragment of the two-dimensional polymeric structure formed by hydrogen-bonded water molecules and acetate anion. Hydrogen bonds are shown with dashed lines.

**Fig. 3.**
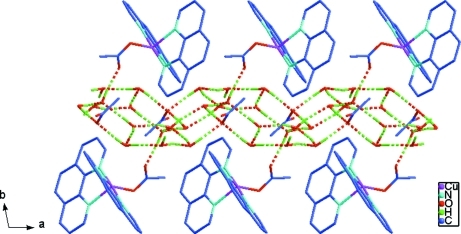
The crystal packing viewed along the c axis. Hydrogen bonds are shown as dashed lines. Hydrogen atoms not involved in hydrogen bonding have been omitted.

## supplementary crystallographic information

### Comment

Construction of supramolecular architectures with intersting physical properties has grown rapidly owing to their potential use as new functional materials. Many intriguing supramolecular assemblies have been prepared by metal coordination or hydrogen bonding interactions. Here, we report a copper(II) complex formed in the reaction of Cu(CH_3_COO)_2_.H_2_O with 1,10-phenanthroline.

Similar to the reported copper(II) complex (Xu *et al.*, 2008), the asymmetric unit of the complex consists of one [Cu(phen)_2_(CH_3_COO)]^+^ complex cation, one acetate anion and seven water molecules. As shown in Fig 1, the central Cu^II^ ion is five coordinate, being bound to four N atoms from two bidentate chelating 1,10-phenanthroline ligands and one O atom from the acetate anion, forming a strongly distorted square-pyramidal geometry. The O1, N1, N4, and N3 atoms are in the equatorial plane, and N2 is in the axial position. The Cu^II^ ion lies 0.2243 (18) Å above the equatorial plane towards N2. The Cu1—N2 bond is significantly longer [2.191 (4) Å] (Table 1), as seen previously [2.1866 (19) Å] (Tu *et al.*, 2008).

In the crystal, hydrogen-bonded water molecules and acetate anion form two-dimensional polymeric structure parallel to (0 1 0) (Fig. 2). The coordination cations are linked to this layer via O-H···O hydrogen bonds (Table 2) between one of the water molecules and coordinated acetate ligand (Fig. 3).

### Experimental

2 ml of aqueous solution of potassium hydroxide (2 mmol, 112.2 mg) were added to a stirred aqueous solution (5 ml) of cupric acetate monohydrate (1 mmol, 199.7 mg) followe by a methanol solution (5 ml) of 1,10-phenanthroline (2 mmol, 396.4 mg). The reaction mixture was and stirred for 4 h. The resultant solution was held at room temperature for ten days, whereupon the blue block-shaped crystals suitable for X-ray diffraction were obtained.

### Refinement

H atoms of the water molecules were found in difference Fourier maps and the O—H distances standardized to 0.85 Å. All other H atoms were placed in geometrically calculated positions (C—H = 0.93-0.96 Å). H atom were allowed to ride on their respective parent atoms, with *U*_iso_(H) = 1.2*U*_eq_(C_phenyl_, O) or 1.5*U*_eq_(C_methyl_).

The SIMU instruction of SHELXL:97 (Sheldrick, 2008) was used to restrain the Uij components of neighboring atoms in the coordinating acetate ligand to be approximately equal with an esd value of 0.1.

### Crystal data

**Table:**

[Cu(C_2_H_3_O_2_)(C_12_H_8_N_2_)_2_](C_2_H_3_O_2_)·7H_2_O	*Z* = 2
*M**_r_* = 668.15	*F*(000) = 698
Triclinic, *P*1	*D*_x_ = 1.400 Mg m^−^^3^
Hall symbol: -P 1	Mo *K*α radiation, λ = 0.71073 Å
*a* = 8.764 (4) Å	Cell parameters from 1605 reflections
*b* = 12.307 (5) Å	θ = 2.5–25.0°
*c* = 15.739 (7) Å	µ = 0.75 mm^−^^1^
α = 103.257 (7)°	*T* = 298 K
β = 102.243 (7)°	Block, blue
γ = 97.606 (7)°	0.42 × 0.38 × 0.32 mm
*V* = 1585.2 (12) Å^3^	

### Data collection

**Table:**

Bruker SMART 1000 CCD area-detector diffractometer	5570 independent reflections
Radiation source: fine-focus sealed tube	3220 reflections with *I* > 2σ(*I*)
graphite	*R*_int_ = 0.040
φ and ω scans	θ_max_ = 25.1°, θ_min_ = 1.4°
Absorption correction: multi-scan (*SADABS*; Sheldrick, 1996)	*h* = −10→10
*T*_min_ = 0.728, *T*_max_ = 0.795	*k* = −13→14
8364 measured reflections	*l* = −18→18

### Refinement

**Table:**

Refinement on *F*^2^	Primary atom site location: structure-invariant direct methods
Least-squares matrix: full	Secondary atom site location: difference Fourier map
*R*[*F*^2^ > 2σ(*F*^2^)] = 0.057	Hydrogen site location: inferred from neighbouring sites
*wR*(*F*^2^) = 0.130	H-atom parameters constrained
*S* = 0.95	*w* = 1/[σ^2^(*F*_o_^2^) + (0.0503*P*)^2^] where *P* = (*F*_o_^2^ + 2*F*_c_^2^)/3
5570 reflections	(Δ/σ)_max_ = 0.001
399 parameters	Δρ_max_ = 0.42 e Å^−^^3^
52 restraints	Δρ_min_ = −0.51 e Å^−^^3^

### Special details

**Table:**

Geometry. All esds (except the esd in the dihedral angle between two l.s. planes) are estimated using the full covariance matrix. The cell esds are taken into account individually in the estimation of esds in distances, angles and torsion angles; correlations between esds in cell parameters are only used when they are defined by crystal symmetry. An approximate (isotropic) treatment of cell esds is used for estimating esds involving l.s. planes.
Refinement. Refinement of F^2^ against ALL reflections. The weighted R-factor wR and goodness of fit S are based on F^2^, conventional R-factors R are based on F, with F set to zero for negative F^2^. The threshold expression of F^2^ > 2sigma(F^2^) is used only for calculating R-factors(gt) etc. and is not relevant to the choice of reflections for refinement. R-factors based on F^2^ are statistically about twice as large as those based on F, and R- factors based on ALL data will be even larger.

### Fractional atomic coordinates and isotropic or equivalent isotropic displacement parameters (Å^2^)

**Table:**

	*x*	*y*	*z*	*U*_iso_*/*U*_eq_	
Cu1	0.76867 (7)	0.90326 (4)	0.21867 (3)	0.0411 (2)	
N1	0.8381 (5)	0.7950 (3)	0.2885 (2)	0.0428 (9)	
N2	0.9810 (4)	1.0118 (3)	0.3166 (2)	0.0410 (9)	
N3	0.7103 (4)	1.0131 (3)	0.1481 (2)	0.0363 (9)	
N4	0.8395 (4)	0.8328 (3)	0.1056 (2)	0.0416 (9)	
O1	0.6008 (4)	0.9292 (3)	0.28563 (19)	0.0505 (8)	
O2	0.4739 (4)	0.7876 (3)	0.1694 (2)	0.0558 (9)	
O3	0.2159 (5)	0.5069 (3)	0.4746 (2)	0.0872 (13)	
O4	0.2102 (5)	0.4656 (3)	0.3301 (2)	0.0790 (11)	
O5	0.3093 (5)	0.6651 (3)	0.6385 (2)	0.0866 (12)	
H30	0.2214	0.6561	0.6532	0.104*	
H29	0.2690	0.6167	0.5879	0.104*	
O6	0.5986 (5)	0.6653 (3)	0.7512 (2)	0.1047 (14)	
H31	0.5088	0.6617	0.7160	0.126*	
H32	0.6565	0.6312	0.7213	0.126*	
O7	0.0874 (4)	0.5649 (3)	0.1938 (2)	0.0842 (12)	
H33	0.1281	0.5270	0.2286	0.101*	
H34	0.1498	0.5661	0.1593	0.101*	
O8	0.2819 (4)	0.5844 (3)	0.0791 (2)	0.0719 (10)	
H36	0.3355	0.6498	0.1092	0.086*	
H35	0.2777	0.5816	0.0242	0.086*	
O9	0.4959 (5)	0.4446 (3)	0.1306 (3)	0.1239 (17)	
H37	0.4355	0.4879	0.1128	0.149*	
H38	0.4911	0.4227	0.1775	0.149*	
O10	0.9190 (4)	0.3319 (3)	0.2593 (2)	0.0804 (11)	
H39	1.0088	0.3710	0.2910	0.096*	
H40	0.8964	0.3689	0.2203	0.096*	
O11	0.7928 (4)	0.4387 (3)	0.1067 (2)	0.0795 (11)	
H41	0.8793	0.4852	0.1345	0.095*	
H42	0.7033	0.4535	0.1133	0.095*	
C1	0.4791 (6)	0.8554 (4)	0.2423 (3)	0.0450 (11)	
C2	0.3383 (6)	0.8525 (4)	0.2816 (3)	0.0719 (14)	
H2A	0.3063	0.9250	0.2895	0.108*	
H2B	0.3659	0.8358	0.3390	0.108*	
H2C	0.2523	0.7947	0.2417	0.108*	
C3	0.7640 (6)	0.6890 (4)	0.2752 (3)	0.0536 (13)	
H3	0.6761	0.6588	0.2263	0.064*	
C4	0.8123 (7)	0.6213 (4)	0.3313 (4)	0.0638 (15)	
H4A	0.7574	0.5473	0.3198	0.077*	
C5	0.9388 (8)	0.6633 (4)	0.4023 (4)	0.0655 (16)	
H5	0.9710	0.6185	0.4404	0.079*	
C6	1.0229 (6)	0.7751 (4)	0.4193 (3)	0.0484 (13)	
C7	0.9670 (5)	0.8385 (3)	0.3599 (3)	0.0383 (11)	
C8	1.0420 (5)	0.9535 (4)	0.3748 (3)	0.0398 (11)	
C9	1.1722 (6)	1.0032 (4)	0.4485 (3)	0.0531 (13)	
C10	1.2401 (7)	1.1171 (5)	0.4607 (4)	0.0696 (16)	
H10	1.3270	1.1532	0.5089	0.084*	
C11	1.1784 (7)	1.1743 (4)	0.4018 (4)	0.0665 (15)	
H11	1.2229	1.2498	0.4092	0.080*	
C12	1.0484 (6)	1.1193 (4)	0.3305 (3)	0.0528 (13)	
H12	1.0068	1.1596	0.2907	0.063*	
C13	1.1577 (7)	0.8286 (5)	0.4935 (3)	0.0684 (17)	
H13	1.1970	0.7869	0.5328	0.082*	
C14	1.2290 (7)	0.9368 (5)	0.5082 (3)	0.0670 (16)	
H14	1.3159	0.9690	0.5573	0.080*	
C15	0.6447 (5)	1.1026 (3)	0.1717 (3)	0.0456 (12)	
H15	0.6223	1.1193	0.2279	0.055*	
C16	0.6083 (6)	1.1721 (4)	0.1156 (3)	0.0523 (13)	
H16	0.5615	1.2340	0.1343	0.063*	
C17	0.6403 (6)	1.1505 (4)	0.0338 (3)	0.0513 (13)	
H17	0.6156	1.1970	−0.0042	0.062*	
C18	0.7112 (5)	1.0573 (4)	0.0067 (3)	0.0425 (11)	
C19	0.7425 (5)	0.9900 (3)	0.0663 (3)	0.0355 (10)	
C20	0.8106 (5)	0.8918 (3)	0.0430 (3)	0.0375 (10)	
C21	0.8427 (5)	0.8604 (4)	−0.0417 (3)	0.0457 (12)	
C22	0.9065 (6)	0.7623 (4)	−0.0606 (3)	0.0626 (14)	
H22	0.9282	0.7372	−0.1164	0.075*	
C23	0.9369 (6)	0.7034 (4)	0.0018 (4)	0.0639 (15)	
H23	0.9800	0.6382	−0.0107	0.077*	
C24	0.9030 (6)	0.7413 (4)	0.0850 (3)	0.0532 (13)	
H24	0.9258	0.7007	0.1277	0.064*	
C25	0.7484 (6)	1.0250 (4)	−0.0786 (3)	0.0571 (14)	
H25	0.7302	1.0693	−0.1189	0.068*	
C26	0.8099 (6)	0.9306 (5)	−0.1011 (3)	0.0595 (14)	
H26	0.8316	0.9108	−0.1574	0.071*	
C27	0.2707 (7)	0.5202 (4)	0.4120 (4)	0.0613 (14)	
C28	0.4211 (7)	0.6073 (5)	0.4293 (4)	0.091 (2)	
H28A	0.4525	0.6486	0.4919	0.136*	
H28B	0.4015	0.6591	0.3928	0.136*	
H28C	0.5044	0.5692	0.4143	0.136*	

### Atomic displacement parameters (Å^2^)

**Table:**

	*U*^11^	*U*^22^	*U*^33^	*U*^12^	*U*^13^	*U*^23^
Cu1	0.0453 (4)	0.0407 (3)	0.0410 (3)	0.0129 (3)	0.0110 (3)	0.0155 (2)
N1	0.045 (3)	0.038 (2)	0.048 (2)	0.0089 (18)	0.013 (2)	0.0147 (18)
N2	0.046 (3)	0.041 (2)	0.039 (2)	0.0131 (18)	0.0132 (19)	0.0120 (18)
N3	0.037 (2)	0.036 (2)	0.037 (2)	0.0099 (17)	0.0103 (18)	0.0087 (16)
N4	0.045 (3)	0.038 (2)	0.043 (2)	0.0141 (18)	0.0104 (19)	0.0107 (18)
O1	0.050 (2)	0.0593 (19)	0.0417 (18)	0.0101 (16)	0.0143 (16)	0.0107 (15)
O2	0.056 (2)	0.060 (2)	0.0471 (19)	0.0071 (16)	0.0128 (17)	0.0082 (16)
O3	0.089 (3)	0.111 (3)	0.068 (3)	0.011 (2)	0.027 (2)	0.034 (2)
O4	0.065 (3)	0.104 (3)	0.065 (3)	0.014 (2)	0.014 (2)	0.018 (2)
O5	0.091 (3)	0.095 (3)	0.076 (3)	0.024 (2)	0.019 (2)	0.028 (2)
O6	0.095 (4)	0.137 (4)	0.075 (3)	0.049 (3)	0.015 (3)	0.005 (3)
O7	0.075 (3)	0.104 (3)	0.081 (3)	0.006 (2)	0.022 (2)	0.042 (2)
O8	0.084 (3)	0.063 (2)	0.060 (2)	0.001 (2)	0.013 (2)	0.0097 (18)
O9	0.092 (4)	0.141 (4)	0.186 (5)	0.059 (3)	0.056 (4)	0.097 (4)
O10	0.072 (3)	0.074 (2)	0.091 (3)	0.004 (2)	0.005 (2)	0.032 (2)
O11	0.057 (3)	0.091 (3)	0.078 (3)	0.001 (2)	0.012 (2)	0.011 (2)
C1	0.044 (3)	0.060 (3)	0.041 (3)	0.016 (2)	0.016 (2)	0.024 (2)
C2	0.062 (3)	0.092 (3)	0.065 (3)	0.013 (3)	0.028 (3)	0.019 (3)
C3	0.057 (4)	0.047 (3)	0.063 (3)	0.018 (3)	0.018 (3)	0.021 (3)
C4	0.081 (5)	0.047 (3)	0.082 (4)	0.027 (3)	0.034 (4)	0.035 (3)
C5	0.096 (5)	0.068 (4)	0.064 (4)	0.048 (4)	0.040 (4)	0.044 (3)
C6	0.056 (4)	0.064 (3)	0.041 (3)	0.032 (3)	0.022 (3)	0.025 (3)
C7	0.042 (3)	0.046 (3)	0.033 (2)	0.017 (2)	0.014 (2)	0.014 (2)
C8	0.031 (3)	0.057 (3)	0.033 (3)	0.012 (2)	0.013 (2)	0.010 (2)
C9	0.046 (4)	0.072 (4)	0.040 (3)	0.018 (3)	0.015 (3)	0.006 (3)
C10	0.052 (4)	0.079 (4)	0.057 (4)	−0.005 (3)	0.009 (3)	−0.006 (3)
C11	0.064 (4)	0.055 (3)	0.068 (4)	−0.010 (3)	0.017 (3)	0.003 (3)
C12	0.056 (4)	0.048 (3)	0.054 (3)	0.002 (3)	0.019 (3)	0.012 (3)
C13	0.078 (5)	0.103 (5)	0.044 (3)	0.054 (4)	0.021 (3)	0.033 (3)
C14	0.064 (4)	0.096 (4)	0.037 (3)	0.035 (4)	0.005 (3)	0.006 (3)
C15	0.050 (3)	0.037 (3)	0.046 (3)	0.007 (2)	0.010 (2)	0.006 (2)
C16	0.053 (4)	0.038 (3)	0.065 (3)	0.014 (2)	0.004 (3)	0.017 (3)
C17	0.047 (3)	0.046 (3)	0.060 (3)	0.004 (2)	0.001 (3)	0.026 (3)
C18	0.035 (3)	0.048 (3)	0.042 (3)	−0.001 (2)	0.004 (2)	0.018 (2)
C19	0.028 (3)	0.040 (3)	0.036 (3)	0.004 (2)	0.005 (2)	0.011 (2)
C20	0.025 (3)	0.044 (3)	0.037 (3)	0.000 (2)	0.004 (2)	0.006 (2)
C21	0.038 (3)	0.053 (3)	0.040 (3)	0.003 (2)	0.010 (2)	0.005 (2)
C22	0.053 (4)	0.075 (4)	0.055 (3)	0.016 (3)	0.018 (3)	0.002 (3)
C23	0.056 (4)	0.061 (3)	0.072 (4)	0.024 (3)	0.022 (3)	−0.001 (3)
C24	0.050 (3)	0.048 (3)	0.064 (3)	0.018 (2)	0.013 (3)	0.014 (3)
C25	0.052 (4)	0.076 (4)	0.048 (3)	0.006 (3)	0.009 (3)	0.032 (3)
C26	0.048 (4)	0.091 (4)	0.042 (3)	0.011 (3)	0.015 (3)	0.022 (3)
C27	0.053 (4)	0.067 (4)	0.063 (4)	0.019 (3)	0.003 (3)	0.021 (3)
C28	0.076 (5)	0.088 (4)	0.105 (5)	−0.005 (4)	0.024 (4)	0.028 (4)

### Geometric parameters (Å, °)

**Table:**

Cu1—N3	1.988 (3)	C5—H5	0.9300
Cu1—N1	1.989 (3)	C6—C7	1.402 (5)
Cu1—O1	2.001 (3)	C6—C13	1.432 (7)
Cu1—N4	2.051 (3)	C7—C8	1.423 (6)
Cu1—N2	2.191 (4)	C8—C9	1.397 (6)
N1—C3	1.328 (5)	C9—C10	1.401 (6)
N1—C7	1.360 (5)	C9—C14	1.435 (6)
N2—C12	1.325 (5)	C10—C11	1.354 (7)
N2—C8	1.355 (5)	C10—H10	0.9300
N3—C15	1.324 (5)	C11—C12	1.386 (7)
N3—C19	1.352 (5)	C11—H11	0.9300
N4—C24	1.326 (5)	C12—H12	0.9300
N4—C20	1.354 (5)	C13—C14	1.339 (7)
O1—C1	1.257 (5)	C13—H13	0.9300
O2—C1	1.242 (5)	C14—H14	0.9300
O3—C27	1.218 (6)	C15—C16	1.383 (5)
O4—C27	1.270 (6)	C15—H15	0.9300
O5—H30	0.8500	C16—C17	1.351 (6)
O5—H29	0.8500	C16—H16	0.9300
O6—H31	0.8501	C17—C18	1.400 (6)
O6—H32	0.8500	C17—H17	0.9300
O7—H33	0.8500	C18—C19	1.396 (5)
O7—H34	0.8500	C18—C25	1.429 (6)
O8—H36	0.8500	C19—C20	1.425 (5)
O8—H35	0.8501	C20—C21	1.399 (5)
O9—H37	0.8500	C21—C22	1.394 (6)
O9—H38	0.8499	C21—C26	1.424 (6)
O10—H39	0.8501	C22—C23	1.352 (6)
O10—H40	0.8499	C22—H22	0.9300
O11—H41	0.8500	C23—C24	1.393 (6)
O11—H42	0.8500	C23—H23	0.9300
C1—C2	1.493 (6)	C24—H24	0.9300
C2—H2A	0.9600	C25—C26	1.350 (6)
C2—H2B	0.9600	C25—H25	0.9300
C2—H2C	0.9600	C26—H26	0.9300
C3—C4	1.387 (6)	C27—C28	1.516 (7)
C3—H3	0.9300	C28—H28A	0.9600
C4—C5	1.343 (7)	C28—H28B	0.9600
C4—H4A	0.9300	C28—H28C	0.9600
C5—C6	1.410 (7)		
			
N3—Cu1—N1	177.12 (15)	C11—C10—C9	119.7 (5)
N3—Cu1—O1	92.58 (13)	C11—C10—H10	120.2
N1—Cu1—O1	89.98 (13)	C9—C10—H10	120.2
N3—Cu1—N4	81.55 (13)	C10—C11—C12	119.4 (5)
N1—Cu1—N4	96.80 (13)	C10—C11—H11	120.3
O1—Cu1—N4	151.73 (14)	C12—C11—H11	120.3
N3—Cu1—N2	98.35 (13)	N2—C12—C11	122.8 (5)
N1—Cu1—N2	79.82 (14)	N2—C12—H12	118.6
O1—Cu1—N2	101.51 (13)	C11—C12—H12	118.6
N4—Cu1—N2	106.69 (14)	C14—C13—C6	122.1 (5)
C3—N1—C7	118.4 (4)	C14—C13—H13	119.0
C3—N1—Cu1	125.9 (3)	C6—C13—H13	119.0
C7—N1—Cu1	115.5 (3)	C13—C14—C9	120.4 (5)
C12—N2—C8	118.3 (4)	C13—C14—H14	119.8
C12—N2—Cu1	132.5 (3)	C9—C14—H14	119.8
C8—N2—Cu1	109.0 (3)	N3—C15—C16	122.3 (4)
C15—N3—C19	118.2 (3)	N3—C15—H15	118.9
C15—N3—Cu1	128.0 (3)	C16—C15—H15	118.9
C19—N3—Cu1	113.8 (3)	C17—C16—C15	120.2 (4)
C24—N4—C20	117.6 (4)	C17—C16—H16	119.9
C24—N4—Cu1	131.1 (3)	C15—C16—H16	119.9
C20—N4—Cu1	111.3 (3)	C16—C17—C18	119.2 (4)
C1—O1—Cu1	106.7 (3)	C16—C17—H17	120.4
H30—O5—H29	91.1	C18—C17—H17	120.4
H31—O6—H32	109.2	C19—C18—C17	117.4 (4)
H33—O7—H34	102.8	C19—C18—C25	118.3 (4)
H36—O8—H35	105.8	C17—C18—C25	124.3 (4)
H37—O9—H38	121.0	N3—C19—C18	122.7 (4)
H39—O10—H40	101.1	N3—C19—C20	116.4 (3)
H41—O11—H42	121.7	C18—C19—C20	121.0 (4)
O2—C1—O1	122.1 (4)	N4—C20—C21	123.4 (4)
O2—C1—C2	120.6 (5)	N4—C20—C19	116.9 (4)
O1—C1—C2	117.2 (4)	C21—C20—C19	119.7 (4)
C1—C2—H2A	109.5	C22—C21—C20	116.6 (4)
C1—C2—H2B	109.5	C22—C21—C26	125.1 (4)
H2A—C2—H2B	109.5	C20—C21—C26	118.2 (4)
C1—C2—H2C	109.5	C23—C22—C21	120.3 (5)
H2A—C2—H2C	109.5	C23—C22—H22	119.9
H2B—C2—H2C	109.5	C21—C22—H22	119.9
N1—C3—C4	122.7 (5)	C22—C23—C24	119.4 (5)
N1—C3—H3	118.7	C22—C23—H23	120.3
C4—C3—H3	118.7	C24—C23—H23	120.3
C5—C4—C3	119.6 (5)	N4—C24—C23	122.6 (4)
C5—C4—H4A	120.2	N4—C24—H24	118.7
C3—C4—H4A	120.2	C23—C24—H24	118.7
C4—C5—C6	120.2 (4)	C26—C25—C18	120.5 (4)
C4—C5—H5	119.9	C26—C25—H25	119.7
C6—C5—H5	119.9	C18—C25—H25	119.7
C7—C6—C5	117.0 (5)	C25—C26—C21	122.3 (4)
C7—C6—C13	118.3 (5)	C25—C26—H26	118.9
C5—C6—C13	124.8 (5)	C21—C26—H26	118.9
N1—C7—C6	122.1 (4)	O3—C27—O4	124.5 (6)
N1—C7—C8	117.7 (4)	O3—C27—C28	120.0 (6)
C6—C7—C8	120.1 (4)	O4—C27—C28	115.6 (5)
N2—C8—C9	122.2 (4)	C27—C28—H28A	109.5
N2—C8—C7	117.8 (4)	C27—C28—H28B	109.5
C9—C8—C7	120.0 (4)	H28A—C28—H28B	109.5
C8—C9—C10	117.6 (5)	C27—C28—H28C	109.5
C8—C9—C14	119.2 (5)	H28A—C28—H28C	109.5
C10—C9—C14	123.3 (5)	H28B—C28—H28C	109.5
			
C3—C4—C5—C6	−0.6 (8)		

### Hydrogen-bond geometry (Å, °)

**Table:**

*D*—H···*A*	*D*—H	H···*A*	*D*···*A*	*D*—H···*A*
O5—H29···O3	0.85	1.90	2.738 (5)	170
O6—H31···O5	0.85	1.92	2.768 (5)	176
O7—H33···O4	0.85	1.96	2.797 (5)	167
O7—H34···O8	0.85	1.91	2.760 (5)	173
O8—H36···O2	0.85	1.86	2.710 (4)	173
O9—H37···O8	0.85	1.98	2.826 (5)	174
O10—H40···O11	0.85	2.23	3.070 (5)	168
O11—H42···O9	0.85	1.89	2.714 (5)	164
O5—H30···O10^i^	0.85	2.03	2.819 (5)	155
O6—H32···O4^i^	0.85	1.94	2.778 (5)	169
O9—H38···O6^i^	0.85	1.94	2.737 (6)	156
O10—H39···O4^ii^	0.85	1.87	2.702 (5)	164
O11—H41···O7^ii^	0.85	1.88	2.724 (5)	170
O8—H35···O11^iii^	0.85	1.97	2.796 (5)	165

Symmetry codes: (i) −*x*+1, −*y*+1, −*z*+1; (ii) *x*+1, *y*, *z*; (iii) −*x*+1, −*y*+1, −*z*.
